# Warming from tropical deforestation reduces worker productivity in rural communities

**DOI:** 10.1038/s41467-021-21779-z

**Published:** 2021-03-11

**Authors:** Yuta J. Masuda, Teevrat Garg, Ike Anggraeni, Kristie Ebi, Jennifer Krenz, Edward T. Game, Nicholas H. Wolff, June T. Spector

**Affiliations:** 1 Global Science, The Nature Conservancy, Arlington, TX USA; 2 School of Global Policy and Strategy, University of California, San Diego, La Jolla, CA USA; 3 Center for Effective Global Action (CEGA), Berkeley, CA USA; 4 Institute for the Study of Labor (IZA), Bonn, Germany; 5 Faculty of Public Health, Mulawarman University, Samarinda, Indonesia; 6 Department of Global Health, University of Washington, Washington, USA; 7 Department of Environmental and Occupational Health Sciences, University of Washington, Washington, USA

**Keywords:** Climate-change adaptation, Economics, Environmental impact, Sustainability

## Abstract

The accelerating loss of tropical forests in the 21st century has eliminated cooling services provided by trees in low latitude countries. Cooling services can protect rural communities and outdoor workers with little adaptive capacity from adverse heat exposure, which is expected to increase with climate change. Yet little is still known about whether cooling services can mitigate negative impacts of heat on labor productivity among rural outdoor workers. Through a field experiment in Indonesia, we show that worker productivity was 8.22% lower in deforested relative to forested settings, where wet bulb globe temperatures were, on average, 2.84 °C higher in deforested settings. We demonstrate that productivity losses are driven by behavioral adaptations in the form of increased number of work breaks, and provide evidence that suggests breaks are in part driven by awareness of heat effects on work. Our results indicate that the cooling services from forests have the potential for increasing resilience and adaptive capacity to local warming.

## Abstract (web summary)

It is expected that tropical deforestation and related increases in heat exposure have negative impacts on labour productivity, but the size of the effect is not well known. Here, the authors show that deforestation reduces productivity by 8.22% in rural Indonesia and causes behavioural adaptation responses like more work breaks.

## Introduction

Trees can provide cooling services via shade and evapotranspiration^1,2^. These ecosystem services are widely recognized in urban areas for mitigating heat island effects^3–6^, but little to no work has examined this benefit in rural areas of low-income countries in the tropics where temperatures already exceed thresholds for human safety^7^. Meanwhile, tropical deforestation continues at a rapid pace^8^, which can lead to local temperature increases over a single season, or even day, that exceed a century of warming under high emissions scenarios^9–12^. This warming affects not only just the most vulnerable community members (i.e., the elderly and the very young) but also the productivity of health workers. Heat may especially be detrimental to outdoor workers because they are often engaged in rigorous physical activity for long durations due to their primary livelihood strategies, such as farming^13^. Yet our understanding of whether trees and the cooling services they provide can increase the well-being and resilience of outdoor workers in such contexts remains limited and therefore undervalued. Researchers have struggled to understand the effects of the cooling services of forests in rural, low-income settings because of lack of data and the presence of multiple unobserved confounding factors, making it challenging to estimate the causal effects of cooling services from trees on worker productivity. We overcome these challenges by conducting a field experiment where we randomly assign workers from rural villages to routine tasks in forested and deforested settings in East Kalimantan, Indonesia.

The focus on rural communities in tropical countries is important for a few reasons. While studies have long argued for the importance of tropical forests for combating global climate change and biodiversity conservation^14,15^, researchers have more recently started highlighting how tropical deforestation can have significant adverse local human health and well-being impacts^16–19^. At the same time, the adverse effects of exposure to hotter temperatures are increasingly seen as an area of concern^20–25^. For rural communities in tropical forest settings where heat and humidity are already high and who are among the most vulnerable to environmental and other shocks^26–29^, deforestation-induced temperature increases through the loss of cooling services may especially be detrimental. Field studies have shown temperatures can be up to 8.3 °C hotter in deforested compared to forested settings^9^. Recent evidence indicates that warming is more extreme as the deforested area becomes larger^11^, and that adverse heat effects may extend up to 50 km beyond deforested sites^30^. Importantly, deforestation is largely driven by human use^8^, and indicates that in deforested areas people are actively working outside. Recent efforts to increase trees on existing agricultural lands and degraded lands provide an opportunity to increase tree cover, and may yield co-benefits to outdoor workers in addition to increasing carbon storage for achieving global climate mitigation goals.

Many studies have examined how exposure to hot environments affects productivity and human health^20,31–37^, but these studies provide limited insight for understanding how heat affects the labor productivity of rural populations facing chronic, deforestation-induced local temperature increases. First, cross-sectional studies suffer from omitted variable bias and are unable to distinguish between the effects of heat and other factors correlated with heat exposure such as income and health. Second, longitudinal studies that overcome omitted variable bias rely on short-run transitory as opposed to longer-run chronic temperature increases. It is challenging to scale estimates from these studies to populations facing deforestation-induced chronic temperature increases because differences in the effects of additional heat exposure can arise due to differences in income (e.g., wealthier individuals may utilize cooling technologies to minimize heat effects) or differences in baseline exposure. For example, a 1 °C increase in overall temperature can have different effects for people living in New Delhi vs. New York either because New Delhi has a higher baseline temperature than does New York or because New Delhi has lower per capita income than New York^38^.

What is also missing from existing studies is an understanding of how people may adapt in real-time to working in hot environments, especially among rural subsistence workers. Behavioral adaptations are one of the first lines of defense against heat^39^, as it allows for reducing internal heat generation and external heat exposure. This is especially important for rural workers in low-income country settings, as they often lack access to the basic infrastructure needed for defenses against excess heat exposure (e.g., air conditioning)^13,29^. Workers may adapt work behaviors in several ways, such as working at a slower pace, spending less time working, or taking more frequent breaks. Survey-based studies have reported outdoor workers in these settings are aware of heat effects^9,34,40,41^, and there are reports that workers are already adapting to hotter thermal environments by shifting the timing, intensity, and type of work they engage in^9^. Importantly, evidence on real-time adaptation to heat is limited, in part due to the lack of availability of high-frequency data. Existing work has focused largely on longer-term adaptations, such as the adoption of cooling technologies^42–44^. More recent work has examined adaptation as changes in behavior from the provision of information, income supplements^45^, or within-day changes in time-use^46^.

Finally, it remains an open question as to whether heat represents a binding physical constraint on productivity, or simply increases the (physical or cognitive) cost of effort. If the former, increasing incentives tied to output are unlikely to increase productivity. If, however, heat increases the cost of effort, then higher incentives are likely to generate higher output.

In our study, we conduct a field experiment that randomly assigns 361 workers from rural communities in East Kalimantan, Indonesia to a 90-min work session in either deforested or forested settings and standard and high piece-rate payment schemes tied to worker output (for details see ‘Methods’). The random assignment allows us to compare productivity differences between two distinct thermal environments while avoiding concerns of omitted variable bias. Unlike past studies, we collect data on worker output, rest-taking behavior, and core body temperature for every minute of the experiment, as well as movements for every second to track physical effort. These data, along with our research design, allow us to evaluate real-time effects on adaptation strategies and output from heat exposure in real-world settings. The field experiment itself is informed by extensive input from study villages, and is designed around one of the most common activities done in and outside of forests—harvesting. Our primary aim is to test whether the loss of trees and their cooling services can have adverse effects via the thermal environment, as this is an increasingly important question given policy efforts to integrate trees onto working lands for climate mitigation and adaptation. We hypothesize that heat exposure from working in deforested areas will lead to significant declines in productivity, and that those receiving larger financial incentives will have greater overall output compared to participants in the standard payment. Further, we hypothesize that those receiving a larger financial incentive will take less breaks even if their rate of production declines, as there is a greater incentive to keep working compared to the standard financial incentive group. Our results indicate that working in deforested settings decreases productivity, but that financial incentives have no effect. Further, we find declines in productivity are driven by rest-taking behavior.

## Results

### Heat exposure due to deforestation reduces productivity

Our results indicate that, even under favorable conditions, working just 90 min in deforested settings adversely impacts productivity (Table 1). Participants working in forested settings were, on average, 8.22% (*p* = 0.0089) more productive than their otherwise equivalent counterparts working in deforested settings (Table 1, Column 1).

**Table 1:** Effect of treatment on productivity, core body temperature, hyperthermia, breaks, and physical activity. Below each coefficient, we report 95% confidence intervals in parenthesis and two-tailed *p* values, respectively. We denote conventional statistical significance as ***p < 0.01, **p < 0.05, *p < 0.1 for two-sided *t* tests. The dependent variable in Column (1) is the log of total output. In Columns (3), (4), and (5), the dependent variable is the inverse hyperbolic sine of, number of minutes with moderate hyperthermia (with moderate hyperthermia (core body temperature exceeding 38.5 ^∘^C), total breaks, and number of minutes spent in moderate-to-vigorous physical activity, respectively. The coefficients can be interpreted as semi-elasticities following appropriate econometric transformations^80^. Core body temperatures are estimated from oral temperatures and heart rate data using a validated algorithm^73^.

	(1)	(2)	(3)	(4)	(5)
	% Output	Core body	% Mins in	% Breaks	% Mins in moderate-to-
		temperature (^∘^C)	hyperthermia		vigorous physical activity
Forested setting	0.0822***	−0.140***	−0.393***	−0.444***	−0.147
	(0.0208, 0.144)	(−0.247, −0.0341)	(−0.598, −0.189)	(−0.626, −0.262)	(−0.366, 0.0717)
	0.00887	0.00982	0.000164	1.67e–06	0.188
High incentive	−0.0266	−0.00642	0.0437	−0.0224	−0.0588
	(−0.0904, 0.0372)	(−0.113, 0.100)	(−0.380, 0.467)	(−0.372, 0.327)	(−0.230, 0.112)
	0.413	0.906	0.840	0.900	0.500
Forest X high incentive	−0.0320	0.0582	−0.0958	0.291	0.0917
	(−0.117, 0.0535)	(−0.0830, 0.199)	(−0.542, 0.351)	(−0.334, 0.916)	(−0.283, 0.466)
	0.462	0.418	0.674	0.362	0.631
Observations	361	361	343	329	329
R-squared	0.371	0.306	0.192	0.232	0.079

Adverse heat effects on productivity likely arise from differences in thermal environments. First, compared to deforested sites, forested sites had, on average, 2.8 °C cooler wet bulb globe temperatures (WBGT) (Supplementary Fig. 1), which is the gold standard measure to assess heat stress^47^ as it captures relative humidity, ambient and black globe temperatures, and wind speed. Second, detailed measurements of core body temperature show that workers in forested settings had significantly lower core body temperatures (Table 1, Column 2), and during the session experienced 39.3% (*p* = 0.0002) lower incidence of moderate hyperthermia (measured as core body temperature exceeding 38.5 °C). Workers in forested settings had median core body temperatures 0.14 °C (*p* = 0.0098) lower than their counterparts in deforested settings (Table 1, Column 3). The range of median core body temperatures recorded in our study is 1.8 °C (min = 36.92 °C, max = 38.72 °C), so the effect is ∼7.8% of the range.

### Workers adapt to heat by taking more breaks

We find that heat-induced productivity declines were driven by adaptive behavior. Participants working in forested settings took 44.4% (*p* = 0.000002) fewer breaks (Table 1, Column 4), and, rather than adjusting the speed or effort of work, rest-taking behavior appears to be the primary mechanism through which we observe productivity declines. We found no difference in moderate-to-vigorous physical activity (MVPA)—a proxy measure for effort calculated from accelerometer data—between participants in forested and deforested settings (Table 1, Column 5). In contrast to prior work^48^, we found that even doubling incentives where participants could earn double the average daily wage rate in 90 min had no effect on heat strain, effort, or productivity, and that the large increase in incentives was unable to induce workers to exert more effort.

Importantly, the thermal environments, which are determined by the amount of tree cover, are likely the key pathway driving productivity declines. Experimental sites were selected with input from villagers to be comparable (e.g., flat, clear from obstructions for the activity, a reasonable distance from the village center). Other factors, such as elevation, are also a negligible concern given our model specification included village fixed effects.

**Fig. 1:**
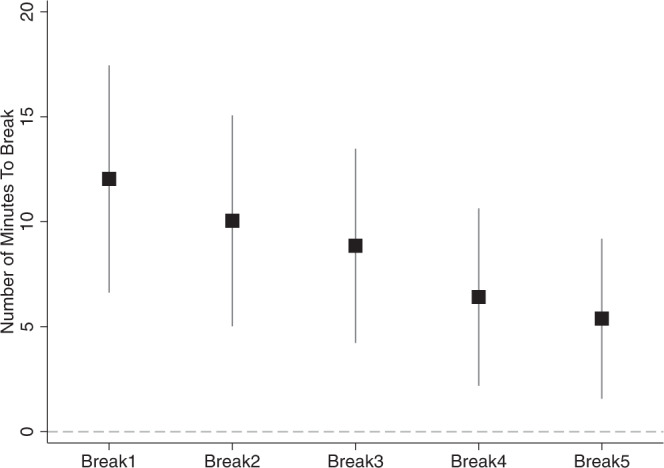
Treatment effect of cumulative time to breaks. This figure shows the treatment effect of being in a forested versus deforested setting on the cumulative time to breaks 1–5. The treatment effect is measured in minutes. The bars around the point estimate denote the 95% confidence interval using robust standard errors. *n* = 361.

One of our primary findings is that participants adapted to working in hotter environments by taking more breaks, as there was no difference in work effort between the two groups. But whether breaks were taken evenly throughout the activity, at the end of the activity, or in some other pattern is important for understanding how behavioral adaptations manifest and can be complemented by other heat protection strategies. We used high-frequency data to estimate the difference in rest-taking behavior between participants in forested and deforested settings over the course of the 90 min experimental session. Figure 1 presents the cumulative time to breaks. We found that in forested settings, participants took the first break, on average, 12 min later than their counterparts in deforested settings. The cumulative effects decreased with each successive break; the difference in time to second break between treatment and control groups was 10 min whereas the difference in time to third break was just under 9 min. However, the differences in time to breaks over successive breaks are not statistically different from each other, though they are individually and jointly significantly different from zero at the 1% level.

### Workers are aware heat exposure affects productivity

Finally, we evaluated how well participants could subjectively assess the effects of heat exposure on their output speed and quality. Health promotion and behavior research^49^ has highlighted how awareness of adverse heat effects is a critical first step for engaging in protective behaviors. To assess whether differences in heat exposure based on work environment affected subjective assessments of heat effects on work speed and output, we asked respondents two different questions: (1) How did heat affect the speed of your work, and (2) How did heat affect the quality of your work. For each question, respondents had three choices: (a) no effect, (b) positive effect, and (c) negative effect. We found that participants working in deforested settings were 15% more likely to report having slower work speed and 12% more likely to report having lower work quality because of heat (Table 2), providing support that participants were aware of adverse effects of heat and is consistent with the evidence of behavioral adaptations.

**Table 2:** Effect of forest treatment on subjective perceptions of the negative effects of heat on productivity. Marginal effects estimated from a multinomial logit regression. The two rows below each coefficient report the 95% confidence intervals in parenthesis and two-tailed *p* values, respectively. We denote conventional statistical significance as ***p < 0.01, **p < 0.05, *p < 0.1 for two-sided *t* tests.

	(1)	(2)
	Output speed	Output quality
Forest setting	−0.152***	−0.121*
	(−0.279, −0.026)	(−0.243, 0.001)
	0.018	0.062
Observations	361	361

## Discussion

Warming from both climate change and deforestation is expected to adversely affect rural communities in low latitude, low-income countries. Dramatic warming from deforestation may exacerbate vulnerability to environmental and other shocks, as many tropical countries are already frequently exceeding thresholds for human safety^7^ and are particularly vulnerable to impacts from climate change^50,51^. Many members of rural communities engage in activities sensitive to heat^13^, such as farming, and have limited adaptive capacity to defend against rising temperatures^29^. Our understanding of how and to what extent these effects will manifest is still emerging. One pathway is through productivity declines of healthy, working populations from increased heat exposure. This population serves as a pillar for the resilience and well-being of families in rural communities, as they are often caretakers, breadwinners, and provide the primary support for vulnerable subgroups, especially absent social protection programs^25,45^. Our study provides unique and timely insights about how deforestation—one of the main drivers of global emissions and a significant source of local warming^9–11,30,52^—can impact productivity through increased heat exposure, and how conserving forests or engaging in tree planting can provide cooling services that can bolster resilience to adverse heat effects.

An important component of our study was the cross-randomization of the level of piece-rate incentives^53^. Surprisingly, we found no evidence that doubling piece-rate incentives affected worker productivity in forested or deforested settings. There are two possible explanations. First, our baseline incentive was already very high and workers were operating at the peak of their physical capacity, so a higher marginal incentive would make little to no difference in effort or output. We conducted extensive validation and testing to ensure financial incentives were, on average, high enough to motivate active participation in the experimental task during the busy agricultural season. Indeed, only two participants out of 402 recruited actively declined to participate, indicating the financial incentive was high enough to have people avoid work and other duties and spend up to 4–5 h in the study. Second, ambient temperatures already approach or exceed safety thresholds for human health and productivity in both forested (average WBGT = 27.41 °C) and deforested (average WBGT = 30.25 °C) settings, and so heat, rather than incentives, represent the binding constraint. Therefore, our study provides evidence on physical constraints to productivity that are unlikely to be overcome through changes in incentives.

Poor households who are liquidity constrained and face incomplete credit and insurance markets are likely unable to smooth consumption, and for these households declines in productivity will decrease resilience to climate change and may potentially lead to poverty traps^28,54,55^. Recent research indicates losses in agricultural output can decrease cognitive performance^25^ and increase suicidal behavior^56^. Despite rural communities having heightened vulnerability to temperature increases compared to their urban counterparts, little work has illuminated whether and to what extent large temperature increases may decrease productivity. Importantly, we are unaware of any work that has studied real-time behavioral adaptations to heat among these populations, especially in local contexts. Given that many of these communities lack access to infrastructure, trees, and their cooling services arguably provide an important and readily available approach to minimizing adverse health effects from increased heat exposure.

Our study documents productivity effects and work adaptations to heat exposure among subsistence agricultural communities, which remains an understudied population^9,34^. We found that even under favorable conditions, working just 90 min in deforested areas, compared to forested areas, decreased productivity by 8.22% for an average 2.84 °C WBGT difference. To put this into context, this is the equivalent effect of increasing ozone exposure by 15 parts per billion (ppb) amongst agricultural workers in the United States^53^ or 13.6 micrograms per cubic meter of PM2.5 exposure amongst pear packers^57^. For reference, the National Ambient Air Quality Standards (NAAQS) in the United States are set at 53 ppb for Ozone and 15 micrograms per cubic meter for PM2.5. Our results indicate that behavioral adaptations in the form of more frequent breaks likely driven by elevated core body temperatures are the primary mechanism for productivity declines. Our experimental design gives us confidence that differences in core body temperatures between participants in forested and deforested areas were elevated largely from external heat exposure rather than internal heat generation, as there were no differences in work effort as measured using high-frequency accelerometer data. Self-reported subjective assessments about whether the external temperature was affecting work speed and quality provide plausible evidence that the greater frequency in break-taking behavior was in part due to participants in deforested areas being aware that their thermal environment was having an adverse effect on work quality and output. Participants are from rural villages in Berau, Indonesia, and as a result, are acclimated to working in local environments. Berau is also emblematic of tropical forest conditions in other countries experiencing land-use pressures from the expansion of agriculture, oil palm, mining, and other activities^58^, with factors such as tenure security shaping the incentives around land use^59,60^. As a result, our results may provide insights into how heat from deforestation is affecting worker productivity of similar populations, of which there is an estimated 800 million people live in or near tropical forests^61^.

Our experiment likely provides conservative estimates of heat effects on productivity. Our experimental protocol limited work time to 90 min and provides favorable working conditions (i.e., access to shade, water, and snacks with participants encouraged to rest *ad libitum*). In contrast, survey data from the same population on work behavior and access to resources important for heat health indicate workers in real-world settings face greater risks of heat strain compared to participants in our study. For instance, time-use data from the same population indicate that, on average, workers spend 6.5 h a day working in agriculture, taking an average of 2.1 breaks during the day^9^. When working in open areas, such as agricultural fields, 90% of outdoor workers have relatively easy access to shade, 94% report wearing protective clothing, and 59% of participants reported having access to water while working^9^. Our experimental activity limits our ability to extrapolate how increased heat exposure would impact productivity for other types of agricultural activities beyond harvesting, which the experimental activity was designed to mimic. Increased heat exposure may lead to different adaptation strategies depending on the farming activity. Activities such as plowing may require greater physical exertion, as, for instance, farmers commonly plowed fields by hand rather than by animal-drawn plows or tractors. In the absence of cooling or other technical interventions, plowing may require more drastic behavioral adaptations given similar thermal environments, while other activities, such as dry direct seeding, may require less physical exertion and thus fewer behavioral adaptations. The study aimed to identify a real-world activity that is done in both forested and deforested settings through extensive testing and in-depth community engagement. Community members at our study sites indicated that such harvesting activities are indeed done in both settings, such as carrying equipment, chopping wood, and harvesting from agricultural fields, which are activities commonly done in other rural communities living in and around forests. However, because tasks are standardized for the purposes of research, our estimates are only measuring the effect of the thermal environment on productivity, not baseline differences by tasks. The randomization of workers to experimental settings alleviates any concern of bias based on prior skill. Future work should empirically test whether and how people adapt differently based on the activity, and assess real-time adaptation strategies over longer time horizons.

The experimental findings speak to broader questions about how rural community lands are being used, and how existing land use patterns may be decreasing resilience to hotter environments. Our study answers the question of how productivity would differ in sites if they were not deforested. This is important because cooling services provided by forested patches are critical for understanding how different land-use patterns are tied to local and global goals. Locally, silvopasture, agroforestry, and other types of agriculture that have the potential to integrate trees into working lands have the potential to also provide cooling services to workers. Yet, there is currently little evidence of these benefits. Globally, natural climate solutions^62–65^, such as the agricultural and land-use practices just mentioned, are a critically important pathways for meeting the nationally determined contributions for the Paris Climate Agreement for many countries. The extent that these practices provide co-benefits to local populations is an important consideration for determining where these practices will advance carbon storage goals and increase the climate resilience of local communities.

While we note several limitations, we also highlight several advances. Prior work has insufficiently documented short-run adaptations; indeed, recent evidence suggests that workers already make marginal changes in their work routines to cope with excess heat exposure^9^. High-frequency data on worker output, core body temperature, and worker adaptation behavior allowed us to examine adjustments workers make in response to excess heat exposure. Our study design also overcomes practical and research design hurdles found in past studies that made estimating the causal effect of heat exposure from deforestation on productivity challenges. Observational studies may suffer from spurious correlation since forest cover loss is associated with economic activity^66–68^ and health effects^16,17^. Using random assignment we overcome these challenges: workers in our setting across deforested and forested settings should be, on average, similar (Supplementary Table 1).

Importantly, our study provides an additional motivation for the significant efforts for global forest restoration and protection, which have, by and large, been rooted in arguments for biodiversity and combating climate change through increased carbon storage^62,69^. Largely missing in these ambitious calls is that strategies, such as wide-scale adoption of agroforestry on pasture or agricultural lands, may provide significant local cooling services that can increase the resilience of subsistence workers to increasing temperatures, and thus have positive spillover effects for their own and their household’s well-being. Identifying strategies that provide climate mitigation and adaptation benefits is critical for achieving global sustainable development and climate change goals, and for increasing climate resilience and adaptive capacity of rural communities in low latitude, low-income countries.

## Methods

The primary methodology in this paper exploits random assignment of workers to forested and deforested settings for a period of 90 min. Within the sample setting we exploit this random assignment to estimate the treatment effect of being in a forested compared to deforested setting. Our analysis examines the causal relationship between exposure to heat in deforested settings on the productivity of workers. Study protocols and outcomes were registered at the American Economic Association’s Randomized Control Trial Registry (Study ID: AEARCTR-0002778) before receiving the data from the experiment. As per our pre-analysis plan, we consider the effect of being in a forested setting, receiving a high piece-rate incentive, and both. All study protocols were approved by the University of Washington Institutional Review Board. Household surveys were drafted and finalized in Microsoft Word 16, and data were entered using CSPro 6.

### Study setting

The study recruited healthy, working adults from ten rural villages in the Berau Regency of East Kalimantan, Indonesia from October 1 to November 6, 2017, the tail end of the dry season. The Berau regency is similar to other tropical forest settings around the world, as the Regency has experienced significant land-use change in the past 20 years from human-driven activities, such as from the expansion of oil palm, agriculture, logging, and mining^58^, which are major industries for the Regency and its population^70^. Its annual rate of forest loss from 2000–2010 is more than 50% higher than the pantropical mean^71^. Daylight hours throughout the year vary little (Supplementary Fig. 2), and communities are engaged in agricultural activities throughout the year. For more on the study setting and population see Aggraeni et al.^72^ and Masuda et al.^9^.

### Sampling

We employed a multiphase random sampling approach to select individuals into our study. We first randomly selected eligible villages, followed by households, and finally eligible individuals within randomly selected households. Inclusion criteria were developed to capture the populations living in and around forests and engaged in manual labor^9,40^. Villages were included if they were (1) on the mainland, (2) had less than 15% of water cover within a 5 km buffer around the village, (3) had less than 5% mangrove cover within a 5 km buffer around the village, (4) was more than 20 km straight-line distance from the regency capitol, and (5) was accessible by road. A 5 km buffer was informed by earlier findings in Kalimantan of deforestation’s effects on perceptions of heat^40^. Thirty-seven out of 113 villages in the Berau Regency met these criteria. We randomly selected five villages above and below the median of intact forest cover (31% of landcover being intact forest in a 5 km buffer) for a total of ten villages from the eligible 37 villages (Supplementary Fig. 3). We randomly sampled from above and below the median of intact forest cover to have even representation of villages embedded more deeply in forests. Finally, individuals were eligible if they were (1) above 21 years old, (2) able to lift more than 10 kilograms, and (3) had no recent or chronic reported respiratory or cardiac issues. The study provided informed consent prior to participation. Participants were offered 20,000 Rupiah for participating in the experimental activity, and, in addition, had the opportunity to earn more based on their performance on the experimental activity.

In total, 363 people were randomized in the experiment (Supplementary Fig. 4). For our primary and secondary analysis, we exclude observations that are missing any key covariates, or are missing sensor data. In total, 361 participants are in our primary analysis on output and breaks, 343 participants are in our evaluation of work effort (i.e., accelerometer data), and 329 participants are in our analysis of core body temperature and minutes in hyperthermia. Randomization checks for those with and without sensors indicate the samples are by and large similar and therefore missing data from sensors is likely random (Tables S2, S3). Participants are, on average, 42 years old, have 6.3 years of education, live in households that consist of ∼4–5 members, and 83% and 73% are farmers and regularly work in forests, respectively. Further information about participants and the study population can be found in Supplementary Table 1 and Aggraeni et al.^72^ and Masuda et al.^9^.

### Experimental design

Enumerators allocated participants via simple randomization to one of four conditions in a 2 × 2 factorial design, where participants selected their experimental assignment out of a container. The experiment involved having participants conduct a generalizable work activity for 90 min. The activity was designed to mimic harvesting activities, and was developed via extensive field testing and input from study villages. For the activity, participants packed 14, 500 *g* bags of dried corn kernels into a backpack, then carried the filled backpack 25 m, and unpacked the bags and created a stacked pile. Participants were instructed to repeat this activity for 90 min, or until participants chose to quit, and were paid a financial incentive for each pile created. Participants were provided water, snacks, and a shaded area to rest *ad libitum*.

The two experimental factors were work setting and the amount of the financial incentive. Participants were assigned to either forest or deforested settings (i.e., an open field) (Supplementary Fig. 5), and to a standard and high piece-rate incentive for each pile the participant created. The standard incentive was set so that, in addition to the participation incentive (20,000 IDR), participants could, on average, earn the daily wage for a day laborer in local villages in 90 min (∼5 USD or 65,000 IDR). The high incentive was set so that, on average, participants could earn approximately double the daily wage rate for a day laborer in local villages in 90 min (∼8 USD or 110,000 IDR). Participants were required to conduct the experimental activity during daylight hours (Supplementary Fig. 2 for daylight hours across the year). Participants were allowed to schedule the day and time of the experimental activity to maximize participation, although within-day randomization suggests this should not be a source of non-random bias (Supplementary Table 1).

During the activity, enumerators collected data on worker output and rest-taking behavior in 1-min intervals. A unit of worker output was recorded whenever a participant collected, carried, and created a pile. We also used Polar® (Polar Inc., Lake Success, NY, USA) and Wahoo Tickr X (Wahoo Fitness, Atlanta, GA, USA) chest band monitors to collect heart rate data for every minute. Heart rate data, along with body temperature data from oral measurements using 53–287 Digital Oral Thermometers (3M Company, Maplewood, MN, USA), were used to estimate 1-min interval core body temperatures using a validated algorithm^73^. This approach uses an extended Kalman filter, along with baseline resting body temperatures and sequential heart rate data, to estimate core body temperatures for every minute^73,74^. We processed the heart rate data by first excluding values outside the physiological range (i.e., <40 or >200 beats per minute) from the raw 1-s interval data, and then averaging heart rates for each minute. For oral temperature measurements, enumerators were trained on how to take oral measurements, as well as where and when measurements should be taken. As such, enumerators took two measurements before the experimental activity with participants in a rested state in a shaded area. These data were checked for extreme outliers, such as those outside physiological limits, and then averaged. We used the Axivity AX3 3-axis accelerometer (Axivity Ltd, New Castle upon Tyne, UK), data logger, to collect data on participant movements, which tracked a participant’s movement in 1-s intervals during the activity. Data loggers were placed on the participants’ dominant hand, and were calibrated before the study. At the end of every day, enumerators downloaded data from the data loggers and checked that data were completely logged for the participant during the time of their experiment. We calculated moderate-to-vigorous activity (MVPA) following Menai et al.^75^, where a participant’s physical activity was counted as being MVPA if the mean 5s-epoch Euclidean Norm Minus One was above 100 mg for at least 1 min^76^. MVPA for the entire session was then calculated by summing the total number of minutes in MVPA for each participant during the 90 min experiment.

Once participants completed the experimental activity, they rested in a shaded area where they were given water and snacks and allowed to rest before completing an additional survey. Survey data collected demographic information, such as age, sex, occupation, educational attainment, and marital status, and also questions about work and time use. We also asked post-activity questions on subjective perceptions of heat on work during the experimental activity. Here, we used two questions from a post experiment survey. One question asked, “How did the heat affect your speed?” The other question asked, “How did the heat affect the quality of your work?” Responses were open-ended, and enumerators have noted whether the responses were positive (e.g., “the heat invigorated me”) or negative (e.g., “the heat slowed me down”). These responses were then coded as the heat (1) having no effect, (2) having a positive effect, and (3) having no effect. In addition, we report descriptive statistics on data from 53–287 Digital Oral Thermometers (3M Company, Maplewood, MN, USA), scales to weigh participants, and 3M QUESTemp WBGT monitors (3M Company, Maplewood, MN, USA) which measured ambient temperature, wet bulb temperature, black globe temperature, relative humidity, and WBGT for experimental sites. Resting oral temperatures were taken before participants started the activity. 3M QUESTemp WBGT monitors were placed at each experimental site for the duration of the data collection period at each village, and were deployed at a height of 1.1 m in the middle of the area where experiments were conducted at each of our forested and deforested sites. The units were randomly assigned a location, and remained deployed at each location until the end of village visits. The units collected data every 5 min, and data were downloaded everyday to a laptop at each location.

Power calculations for a 2 × 2 factorial experiment with one observation per participant at *α* = 0.05 and power = 0.80 indicate that for a conservative sample size (${\mathrm{Cohen}}^{\prime} {\mathrm{s}}f=0.14$), 400 individual participants are needed. Power calculations were done to estimate the interaction effect, and thus have sufficient power to estimate main effects given the efficiency gains from employing a factorial design.

### Statistical methods

We estimated the average treatment effect of deforested conditions on productivity using ordinary least squares regressions with indicator variables for the two experimental factors, as well as an interaction of the two indicator variables to account for the factorial design. We employed clustered robust standard errors at the individual level, which is the unit of randomization^77^, and village fixed effects to account for unobserved heterogeneity across villages. We present fully adjusted models that include baseline covariates for age, an indicator variable for female, years of education, an indicator variable if the respondent indicated their primary occupation was farming, an indicator for regularly collecting firewood for the household which reflects familiarity with the experimental activity, an indicator for regularly working in the forest, self-assessed health status, body mass index, an indicator variable if the activity was conducted past noon to account for diurnal trends, and an indicator variable if it rained during the activity. We included these covariates to increase the precision of estimates in case baseline covariates are correlated with the outcome, as well as to correct for any imbalance in baseline covariates between the experimental groups^78,79^. Doing so can increase statistical power and efficiency by subtracting explained variation for linear models^78,79^. We show in the supplemental materials (Supplementary Table 4) that our findings are similar with and without these covariates. Finally, our analysis on subjective perceptions of heat on work used two questions from a post experiment survey and employed a multinomial logit model using the same specification discussed above, and then estimated marginal effects holding variables at their means.

For analysis pertaining to Fig. 1, we calculated the cumulative time lapsed in the experiment before each break is taken. If a study participant had not taken a given break, we coded them as if they had taken that break at the end of the study which means the time to each break is capped at the total study duration of 90 min. It is possible that the participant may not have taken a break until beyond the 90 min mark if the study had continued; therefore, our effects represent a lower bound on the effects of working in a forested setting on productivity. We limit our analysis to the first five breaks taken, but our analysis can easily be extended to a higher number of breaks. The coefficient reported for the effect of treatment on cumulative time to each break is estimated independently in a separate regression.

All statistical analyses were performed with Stata version 14 (Stata Corp, College Station, TX, USA).

### Reporting summary

Further information on research design is available in the Nature Research Reporting Summary linked to this article.

## Supplementary information

Supplementary Information

Reporting Summary

## Data Availability

The datasets generated during and/or analyzed during the current study are available upon reasonable request.

## Code availability

The code for the analyses is available upon reasonable request.
