# Supplementary material for: Warming from tropical deforestation reduces worker productivity in rural communities
Source: Nat Commun. 2021 Mar 11;12:1601. doi: 10.1038/s41467-021-21779-z (PMC7952402; doi:10.1038/s41467-021-21779-z)
Supplement: Supplementary file 1 — Supplementary Information [file 41467_2021_21779_MOESM1_ESM.pdf]

Supplementary Information for:

Warming from Tropical Deforestation Reduces Worker Productivity in Rural Communities

Figure S1: Average and time series wet bulb globe temperature values by experimental site. The plot represents elapsed time for participants during the experimental activity. Shaded regions represent 95% confidence intervals calculated from 4,718 observations. Mean WGBT for forested and deforested settings are 27.41°C and 30.25°C, respectively. Deforested sites had differences that were statistically significant from forested sites along all these variables.

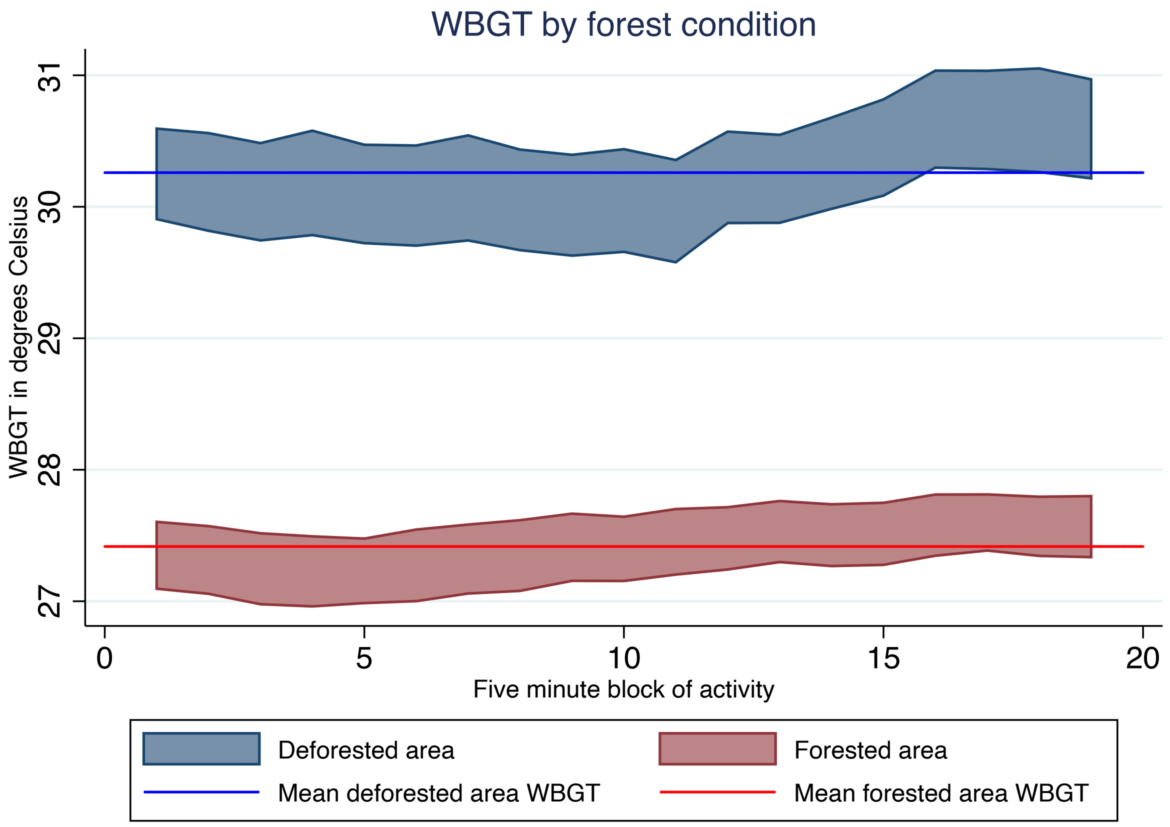

Figure S2: Daylight hours for the Berau Regency, Indonesia.

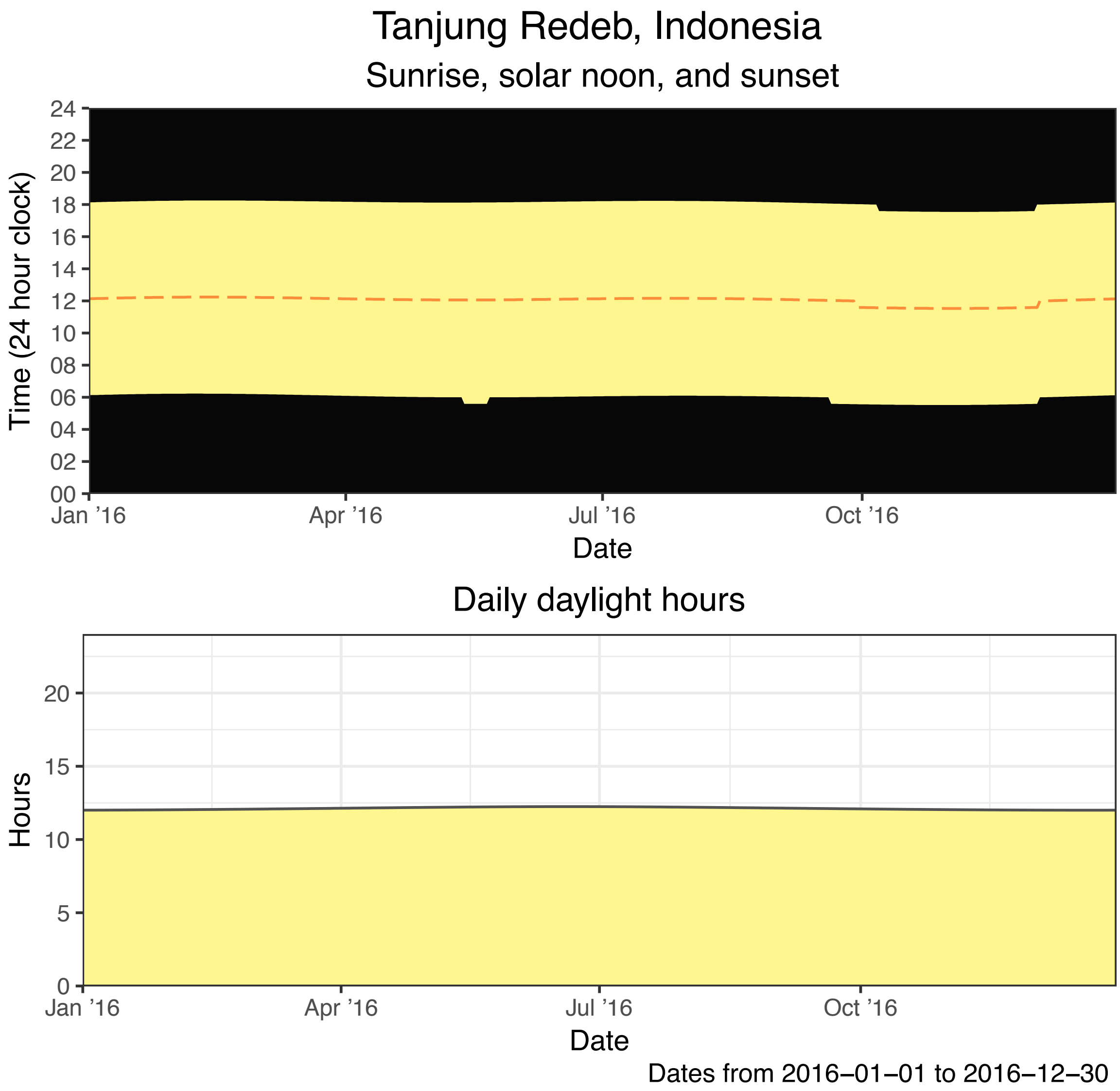

Figure S3: Study villages and canopy cover[a]

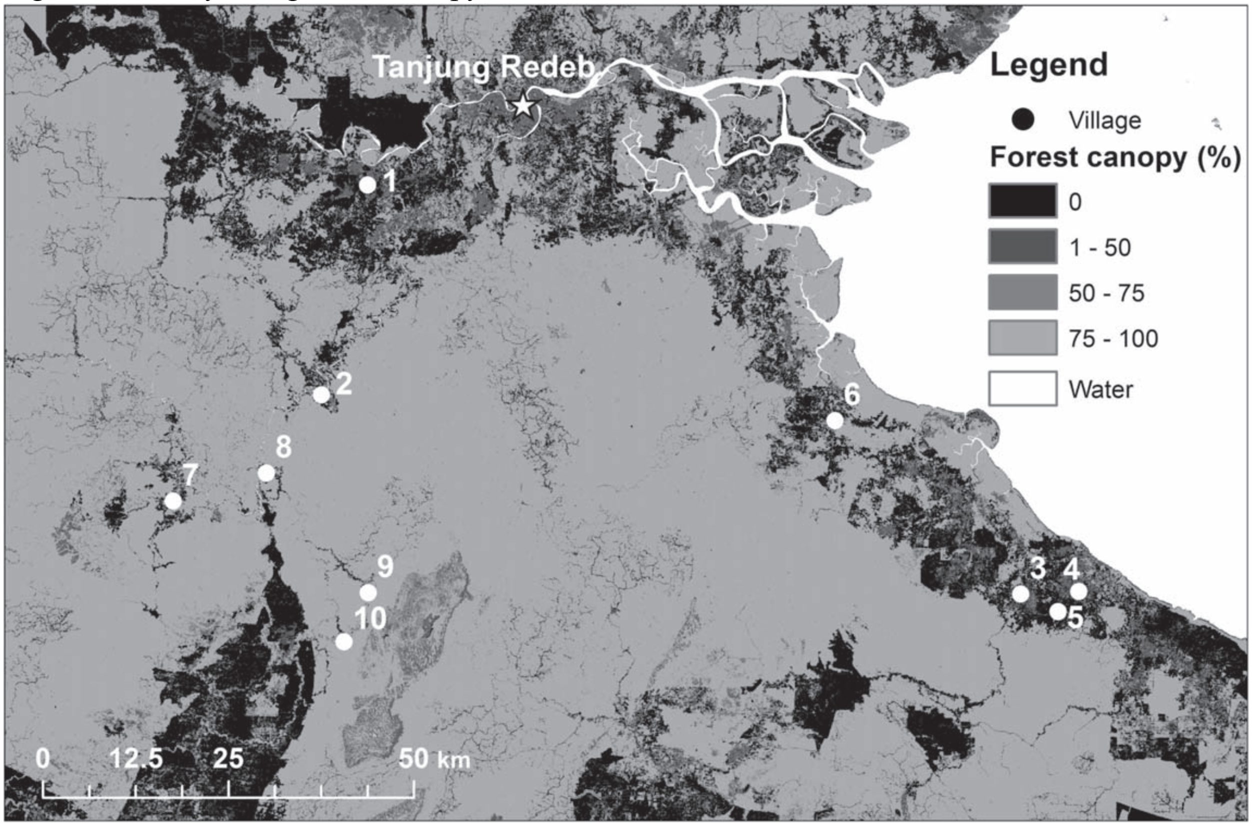

[a]Figure created by Nicholas Wolff.

Figure S4: Sample selection, randomization, and sample size diagram.

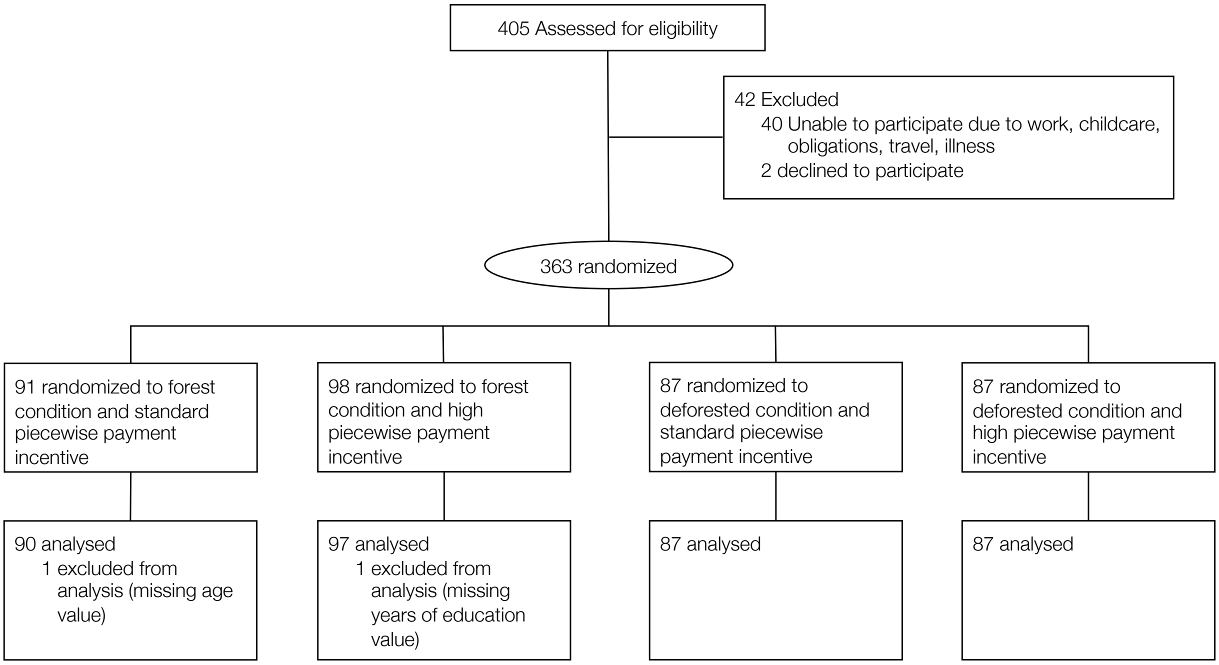

Figure S5: Examples of experimental setting sites.

Panel A: Forested setting

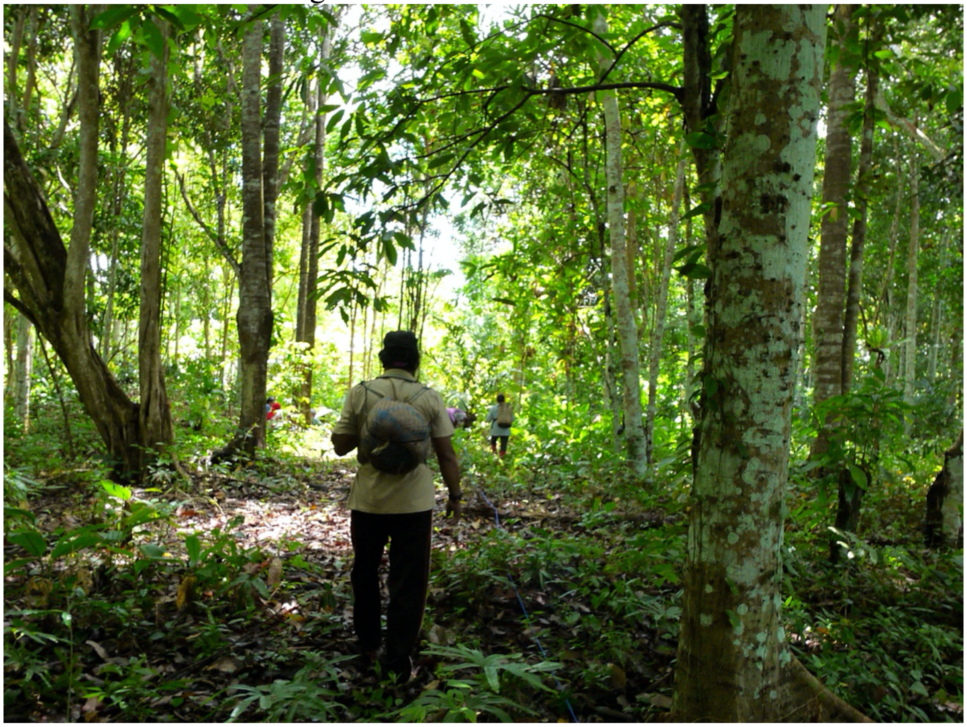

Panel B: Deforested setting

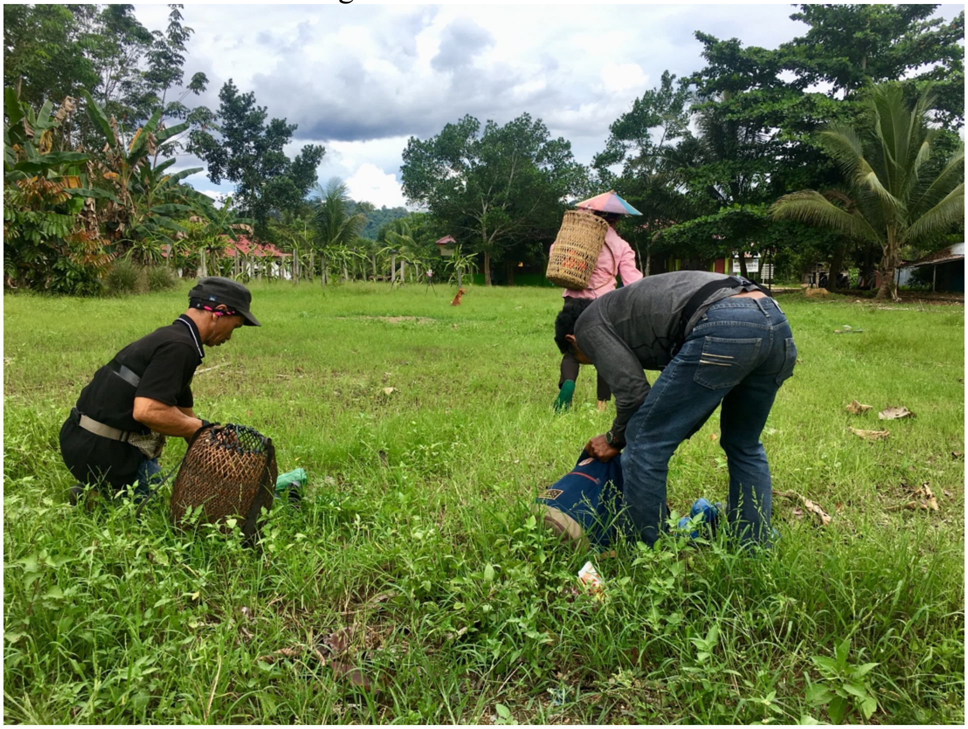

Table S1: Descriptive statistics by experimental group[a]

	Forest-Standard	Forest-High	Open area-Standard	Open area-High	p-value[b]
Age	41	42	40	44	0.091
	(12)	(10)	(11)	(11)	
Female (%)	34	63	39	53	0.000
	(48)	(48)	(49)	(50)	
Years of education	6.2	6.1	7.0	5.8	0.133
	(3.8)	(3.2)	(3.5)	(3.6)	
Firewood collector (%)	63	69	62	64	0.710
	(49)	(46)	(49)	(48)	
Works in forest (%)	70	69	83	62	0.028
	(46)	(38)	(38)	(49)	
Farmer (%)	80	88	78	86	0.243
	(40)	(33)	(42)	(35)	
Individual health assessment[c]	3.1	3.1	3.1	3.1	0.906
	(0.75)	(0.86)	(0.81)	(0.84)	
Body mass index	23	25	23	24	0.067
	(3.8)	(4.3)	(3.8)	(3.8)	
Resting oral temperature (°C)	36.8	36.8	36.7	36.7	0.19
	(0.29)	(0.41)	(0.40)	(0.38)	
Household size	4.6	4.4	4.5	4.5	0.772
	(1.5)	(1.5)	(1.5)	(1.4)	
Log income (IDR)[d]	16	16	16	16	0.582
	(2.2)	(1.9)	(2.8)	(2.7)	
Log household assets (IDR)[d]	17	17	17	17	0.173
	(0.98)	(1.2)	(1.1)	(1.0)	
n	91	98	87	87	

[a] Standard deviations are presented in parentheses.
[b] Dichotomous variables present p-values for Pearson's χ^2, while for continuous variables present p-values for F-tests from a one-way ANOVA.
[c] Respondents were asked to assess their overall health status on a five point scale asking, "Would you say that, in general, your health is excellent, very good, good, fair, or poor?"
[d] Income includes both farm and non-farm income. Household assets include productive (e.g., plows, machetes) and non-productive household assets (e.g., televisions, radios).

Table S2: Descriptive statistics for sample with and without all sensor data[a]

	Does not have all sensor data	Has all sensor data	Difference	p-value
Age	43	42	1.3	0.504
	(11)	(11)		
Female (%)	39	49	-9.1	0.285
	(7.9)	(2.8)		
Years of education	6.2	6.3	-0.13	0.830
	(3.4)	(3.6)		
Firewood collector (%)	66	65	1.2	0.886
	(7.7)	(2.7)		
Works in forest (%)	82	73	8.7	0.250
	(6.2)	(2.4)		
Farmer (%)	79	84	-4.7	0.459
	(6.6)	(2.0)		
Individual health assessment[b]	3.1	3.2	-0.049	0.728
	(0.73)	(0.82)		
Body mass index	25	24	0.81	0.235
	(4.3)	(3.9)		
Household size	4.3	4.5	-0.19	0.440
	(1.4)	(1.5)		
Log income (IDR)	16	16	-0.19	0.142
	(2.9)	(2.4)		
Log household assets (IDR)[c]	17	17	0.27	0.648
	(1.2)	(1.1)		
n	38	325		

[a] Standard deviations are presented in parentheses.
[b] Respondents were asked to assess their overall health status on a five point scale asking, "Would you say that, in general, your health is excellent, very good, good, fair, or poor?"
[c] Income includes both farm and non-farm income. Household assets include productive (e.g., plows, machetes) and non-productive household assets (e.g., televisions, radios).
*** $p<0.01$, ** $p<0.05$, * $p<0.10$ represent significance for two-sided t-tests for continuous variables and equality of proportions test for dichotomous variables.

Table S3: Descriptive statistics for sample with and without core body temperature (CBT) data[a]

	Does not have CBT data	Has CBT data	Difference	p-value
Age	42	42	-0.66	0.744
	(9.86)	(11.00)		
Female (%)	41	48	-7.7	0.404
	(8.6)	(2.7)		
Years of education	6.5	6.3	0.22	0.740
	(3.4)	(3.6)		
Firewood collector (%)	66	65	1.0	0.912
	(8.4)	(2.6)		
Works in forest (%)	84	73	12	0.155
	(6.4)	(2.4)		
Farmer (%)	75	84	-9.0	0.194
	(7.6)	(2.0)		
Individual health assessment[b]	3.1	3.2	-0.094	0.531
	(0.76)	(0.8)		
Body mass index	25	24	0.97	0.185
	(4.4)	(3.9)		
Household size	4.3	4.5	-0.23	0.388
	(1.4)	(1.5)		
Log income (IDR)	16	16	-0.42	0.354
	(3.1)	(2.3)		
Log household assets (IDR)[c]	17	17	0.34*	0.088
	(1.3)	(1.1)		
n	32	331		

[a] Standard deviations are presented in parentheses.

[b] Respondents were asked to assess their overall health status on a five point scale asking, "Would you say that, in general, your health is excellent, very good, good, fair, or poor?"

[c] Income includes both farm and non-farm income. Household assets include productive (e.g., plows, machetes) and non-productive household assets (e.g., televisions, radios).

*** p<0.01, ** p<0.05, * p<0.10 represent significance for two-sided t-tests for continuous variables and equality of proportions test for dichotomous variables

Table S4: Effects of treatment on productivity, breaks, movement, core body temperatures, and hyperthermia without controls. For all coefficients, we report robust standard errors in parenthesis denoting conventional statistical significance as *** p<0.01, ** p<0.05, * p<0.1 for two-sided t-tests. The dependent variable in Column (1) is the log of total output. In Columns (3), (4), and (5), the dependent variable is the inverse hyperbolic sine of number of minutes with moderate hyperthermia (core body temperature exceeding 38.5C), total breaks, and number of minutes spent in moderate-to-vigorous physical activity respectively. The coefficients can be interpreted as semi-elasticities following appropriate econometric transformations (Bellemare & Wichman, 2019).

**% output**	**Coefficient**	**95% CI**	***p*-value**
Forested Setting	0.094**	(0.019, 0.169)	0.014
High Incentive	-0.032	(-0.110, 0.046)	0.421
Forest X High	-0.062	(-0.162, 0.038)	0.227
Observations		363	
R-squared		0.029	
**Core body temperature (°C)**			
Forested Setting	-0.112*	(-0.226, 0.001)	0.053
High Incentive	0.0401	(-0.080, 0.160)	0.512
Forest X High	0.0807	(-0.078, 0.239)	0.318
Observations		331	
R-squared		0.025	
**% hyperthermia (mins)**			
Forested Setting	-0.365***	(-0.774, -0.103)	0.011
High Incentive	0.0525	(-0.378, 0.534)	0.737
Forest X High	0.0123	(-0.469, 0.562)	0.859
Observations		331	
R-squared		0.032	
**% breaks**			
Forested Setting	-0.435***	(-0.916, -0.190)	0.011
High Incentive	0.060	(-0.308, 0.465)	0.640
Forest X High	0.234	(-0.253, 0.788)	0.576
Observations		363	
R-squared		0.036	
**% MVPA (mins)**			
Forested Setting	-0.128	(-0.351, 0.089)	0.404
High Incentive	-0.0299	(-0.198, 0.145)	0.902
Forest X High	0.0476	(-0.257, 0.376)	0.708
Observations		345	
R-squared		0.004	
