# Supplementary material for: Warming from tropical deforestation reduces worker productivity in rural communities
Source: Nat Commun. 2021 Mar 11;12:1601. doi: 10.1038/s41467-021-21779-z (PMC7952402; doi:10.1038/s41467-021-21779-z)
Supplement: Supplementary file 2 — Reporting Summary [file 41467_2021_21779_MOESM2_ESM.pdf]

nature research

Corresponding author(s): Yuta J. Masuda

Last updated by author(s): Oct 2, 2020

Reporting Summary

Nature Research wishes to improve the reproducibility of the work that we publish. This form provides structure for consistency and transparency in reporting. For further information on Nature Research policies, see our Editorial Policies and the Editorial Policy Checklist.

Statistics

For all statistical analyses, confirm that the following items are present in the figure legend, table legend, main text, or Methods section.

n/a	Confirmed	
☐	☒	The exact sample size (*n*) for each experimental group/condition, given as a discrete number and unit of measurement
☐	☒	A statement on whether measurements were taken from distinct samples or whether the same sample was measured repeatedly
☐	☒	The statistical test(s) used AND whether they are one- or two-sided<br>*Only common tests should be described solely by name; describe more complex techniques in the Methods section.*
☐	☒	A description of all covariates tested
☐	☒	A description of any assumptions or corrections, such as tests of normality and adjustment for multiple comparisons
☐	☒	A full description of the statistical parameters including central tendency (e.g. means) or other basic estimates (e.g. regression coefficient) AND variation (e.g. standard deviation) or associated estimates of uncertainty (e.g. confidence intervals)
☐	☒	For null hypothesis testing, the test statistic (e.g. *F*, *t*, *r*) with confidence intervals, effect sizes, degrees of freedom and *P* value noted<br>*Give P values as exact values whenever suitable.*
☒	☐	For Bayesian analysis, information on the choice of priors and Markov chain Monte Carlo settings
☒	☐	For hierarchical and complex designs, identification of the appropriate level for tests and full reporting of outcomes
☐	☒	Estimates of effect sizes (e.g. Cohen's *d*, Pearson's *r*), indicating how they were calculated

Our web collection on statistics for biologists contains articles on many of the points above.

Software and code

Policy information about availability of computer code

Data collection	Surveys were drafted and finalized in Microsoft Word 16, printed, then data were double-entered in CSPro 6.
Data analysis	All statistical analyses were conducted on Stata version 14.

For manuscripts utilizing custom algorithms or software that are central to the research but not yet described in published literature, software must be made available to editors and reviewers. We strongly encourage code deposition in a community repository (e.g. GitHub). See the Nature Research guidelines for submitting code & software for further information.

Data

Policy information about availability of data

All manuscripts must include a data availability statement. This statement should provide the following information, where applicable:

- Accession codes, unique identifiers, or web links for publicly available datasets
- A list of figures that have associated raw data
- A description of any restrictions on data availability

The datasets in this study are available from the corresponding author upon reasonable request.

Field-specific reporting

Behavioural & social sciences study design

All studies must disclose on these points even when the disclosure is negative.

Study description	We report on a field experiment that utilizes a 2x2 factorial design, where the two experimental factors were work setting (forested vs. deforested setting) and the amount of the financial incentive (standard vs. high incentive payment). 361 workers from rural communities in East Kalimantan, Indonesia were randomly assigned to one of four experimental arms to a 90 minute work session. We collected quantitative data via surveys and high frequency sensors on worker output, movement, core body temperatures, and rest taking behavior, as well as self-reported assessments of worker performance and assessments of the thermal environment.
Research sample	The study recruited healthy, working adults from 10 rural villages in the Berau Regency of East Kalimantan, Indonesia. The Berau regency is similar to other tropical forest settings around the world, as the Regency has experienced significant land use change in the past 20 years from human-driven activities, such as from the expansion of oil palm, agriculture, logging, and mining, which are major industries for the Regency and its population. We employed a multiphase random sampling approach to select individuals into our study. We first randomly selected eligible villages, followed by households, and finally eligible individuals within randomly selected households. Inclusion criteria were developed to capture the populations living in and around forests and engaged in manual labor, and adhered requirements outlined in human subjects research review. Individuals were eligible if they were 1) above 21 years old, 2) able to lift more than 10 kilograms, and 3) had no recent or chronic reported respiratory or cardiac issues. The study provided informed consent prior to participation. Participants were offered 20,000 Rupiah for participating in the experimental activity, and, in addition, had the opportunity to earn more based on their performance on the experimental activity.<br>In total, 363 people were included in our study. Forty-seven percent of participants were female and 83 percent were farmers. On average, participants were 42 years old, had 6.3 years of education, and had 4.4 household members. Given our sampling approach, the participants our study are representative of healthy, adult outdoor workers in rural communities in Berau, Indonesia. This population is our target population because they are the primary breadwinners for households, and often are caretakers and provide primary support for vulnerable subgroups. As a result, any productivity declines from heat is important for their own and their household's well-being.
Sampling strategy	Power calculations for a 2 x 2 factorial experiment with one observation per participant at α = 0.05 and power = 0.80 indicate that for a conservative sample size (Cohen's f = 0.14), 400 individual participants are needed. Power calculations were done to estimate the interaction effect, and thus have sufficient power to estimate main effects given the efficiency gains from employing a factorial design. As stated above, we used a multiphase sampling procedure to recruit participants.
Data collection	Our study utilized household and individual surveys and environmental and personal sensors. During the activity enumerators collected data on worker output and rest taking behavior in one minute intervals. A unit of worker output was recorded whenever a participant collected, carried, and created a pile. We also used Polar ® (Polar Inc., Lake Success, NY) and Wahoo Tickr X (Wahoo Fitness, Atlanta, GA) chest band monitors to collect heart rate data for every minute. Heart rate data, along with core body temperature data from oral measurements, were used to estimate one minute interval core body temperatures using a validated algorithm. To do so, we processed the heart rate data by first excluding values outside the physiological range (i.e., < 40 or > 200 beats per minute) from the raw one second interval data, and then averaging heart rates for each minute. We used the Axivity AX3 3-axis accelerometer (Axivity Ltd, New Castle upon Tyne, UK) data logger to collect data on participant movements, which tracked a participant's movement in one second intervals during the activity. Data loggers were placed on the participants' dominant hand, and were calibrated before the study. At the end of everyday, enumerators downloaded data from the data loggers and checked that data were completely logged for the participant during the time of their experiment.<br><br>Once participants completed the experimental activity, they rested in a shaded area where they were given water and snacks and allowed to rest before completing an additional survey with just the enumerator. Survey data collected demographic information, such as age, sex, occupation, educational attainment, and marital status, and also questions about work and time use. We also asked post-activity questions on subjective perceptions of heat on work during the experimental activity. Our study used two questions from a post-experiment survey. One question asked, "How did the heat affect your speed?" The other question asked, "How did the heat affect the quality of your work?" Responses were open-ended, and enumerators were noted whether the responses were positive (e.g., "the heat invigorated me") or negative (e.g., "the heat slowed me down"). These responses were then coded as the heat (1) having no effect, (2) having a positive effect, and (3) having no effect. In addition, we report descriptive statistics on data from 53-287 Digital Oral Thermometers (3M Company, Maplewood, MN), scales to weigh participants, and 3M QUESTemp WBGT monitors (3M Company, Maplewood, MN) which measured ambient temperature, wet bulb temperature, black globe temperature, relative humidity, and WBGT for experimental sites. Resting oral temperatures were taken before before participants started the activity. 3M QUESTemp WBGT monitors were placed at each experimental site for the duration of the data collection period at each village, and were deployed at a height of 1.1m in the middle of the area where experiments were conducted at each of our forested and deforested sites. units were randomly assigned a location, and remained deployed at each location until the end of village visits. The units collected data every five minutes, and data were downloaded everyday to a laptop at each location.<br><br>Data were collected by author Ike Anggraeni and a team of local enumerators, and the team was not not blinded to the experimental conditions or research hypotheses.
Timing	Data collection occurred between October 1 to November 6, 2017, the tail end of the dry season.
Data exclusions	Data for two participants were excluded from our analysis due to missing values.
Non-participation	The study recruited 405 eligible individuals in randomly selected villages and households. Forty individuals were unable to participate due to work, childcare, obligations, travel, illness, and two individuals declined to participate, leaving 363 individuals (90%) that underwent randomization.
Randomization	All participants were randomly allocated to their experimental groups.

Reporting for specific materials, systems and methods

We require information from authors about some types of materials, experimental systems and methods used in many studies. Here, indicate whether each material, system or method listed is relevant to your study. If you are not sure if a list item applies to your research, read the appropriate section before selecting a response.

Materials & experimental systems

n/a	Involved in the study
☒	☐ Antibodies
☒	☐ Eukaryotic cell lines
☒	☐ Palaeontology and archaeology
☒	☐ Animals and other organisms
☐	☒ Human research participants
☐	☒ Clinical data
☒	☐ Dual use research of concern

Methods

n/a	Involved in the study
☒	☐ ChIP-seq
☒	☐ Flow cytometry
☒	☐ MRI-based neuroimaging

Human research participants

Policy information about studies involving human research participants

Population characteristics	See above.
Recruitment	See above about participant recruitment.
Ethics oversight	University of Washington Institutional Review Board

Note that full information on the approval of the study protocol must also be provided in the manuscript.

Clinical data

Policy information about clinical studies

All manuscripts should comply with the ICMJEguidelines for publication of clinical research and a completedCONSORT checklist must be included with all submissions.

Clinical trial registration	AEARCTR-0002778
Study protocol	American Economic Association's Randomized Control Trial Registry
Data collection	Berau Regency of East Kalimantan, Indonesia from October 1 to November 6, 2017
Outcomes	Primary and secondary outcome measures were pre-defined during the pre-study and survey development period. A scoping visit was conducted by the data collection team to assess the appropriateness of these measures. Study protocols and outcomes were registered before data were received.
